# Specific chromatin states and m6A modifications are associated with mRNA mobility *in planta*

**DOI:** 10.1093/hr/uhae101

**Published:** 2024-04-08

**Authors:** Xiaojun Li, Veli Vural Uslu, Ying Chen, Xiao Han, Alexandre Berr, Wenna Zhang, Yihan Dong

**Affiliations:** Beijing Key Laboratory of Growth and Developmental Regulation for Protected Vegetable Crops, China Agricultural University, Beijing 100193, China; RLP AgroScience GmbH, Neustadt an der Weinstraße 67435, Germany; MAPS, Center for Organismal Studies, Heidelberg University, Heidelberg 69120, Germany; Beijing Key Laboratory of Growth and Developmental Regulation for Protected Vegetable Crops, China Agricultural University, Beijing 100193, China; College of Biology Science and Engineering, Fuzhou University, Fuzhou, China; Institut de Biologie Moléculaire des Plantes, Centre National de la Recherche Scientifique, UPR 2357, Université de Strasbourg, Strasbourg, France; Beijing Key Laboratory of Growth and Developmental Regulation for Protected Vegetable Crops, China Agricultural University, Beijing 100193, China; Institut de Biologie Moléculaire des Plantes, Centre National de la Recherche Scientifique, UPR 2357, Université de Strasbourg, Strasbourg, France

Dear Editor,

A growing body of research has increasingly shed light on the mobility and systemic signaling roles of messenger RNAs (mRNAs). The field of plant grafting, especially horticultural crops, has provided scientists with the unique opportunity to identify thousands of mobile mRNAs. This, in turn, gives rise to a tantalizing question: What factors govern mRNA mobility?

One prevailing model suggests that RNA mobility hinges on sequence-specific motifs, such as the epitranscriptomic RNA modification 5-methylcytosine (m5C) [1]. Yang et al. demonstrated a positive correlation between the number of m5C sites and mRNA mobility. Furthermore, they characterized two mRNAs influenced by m5C modification, namely *TUMOR CONTROLLED TRRANSLATION PRPTEIN 1* (*TCTP1*) and *HEAT SHOCK* homologous protein 70.1 (*HSC70.1*). Importantly, the mobility of these mRNAs is governed by m5C modification and appears to be independent of their mRNA abundance [1]. It is worth noting, however, that m5C-modified mRNAs constitute only about 10% of the total mobile mRNA pool [1], suggesting the involvement of other factors besides m5C.

An alternative computational model suggests that mRNA mobility may primarily hinge on RNA abundance rather than sequence specificity [2]. Recent research provides an intriguing insight: m5C peaks tend to be concentrated in chromatin regions devoid of the repressive histone mark H3K27me3 [3]. This implies a positive association between m5C-modified mRNAs and active transcription. In an attempt to reconcile these divergent models, we have revisited experimental data on mobile mRNAs. Our findings suggest a refined perspective, indicating that mobile mRNAs are linked with specific chromatin states and predominantly associated with another RNA epigenetic mark, N^6^-methyladenosine (m6A) modifications.

The chromatin status has been categorized into nine distinct states, each associated with varying patterns of gene expression [4]. Our analysis revealed that genes coding for mobile mRNAs [5] and m5C-modified mRNAs [1] displayed significant enrichments in chromatin states 1 (CS-1) and 2 (CS-2) (Fig. 1A). CS-1 is characterized by the presence of various active histone marks, including H3K4me3, H3K9ac, and H3K36me3, suggesting relatively active transcription without necessarily implying an absolute abundance of transcripts [4]. CS-2 represents a bistable chromatin state marked by both the active histone mark H3K4me3 and the repressive one H3K27me3 [4]. CS-2 genes exhibited low-to-medium expression level, suggesting the association of mobile mRNAs with specific chromatin states rather than absolute mRNA abundance.

**Figure 1:**
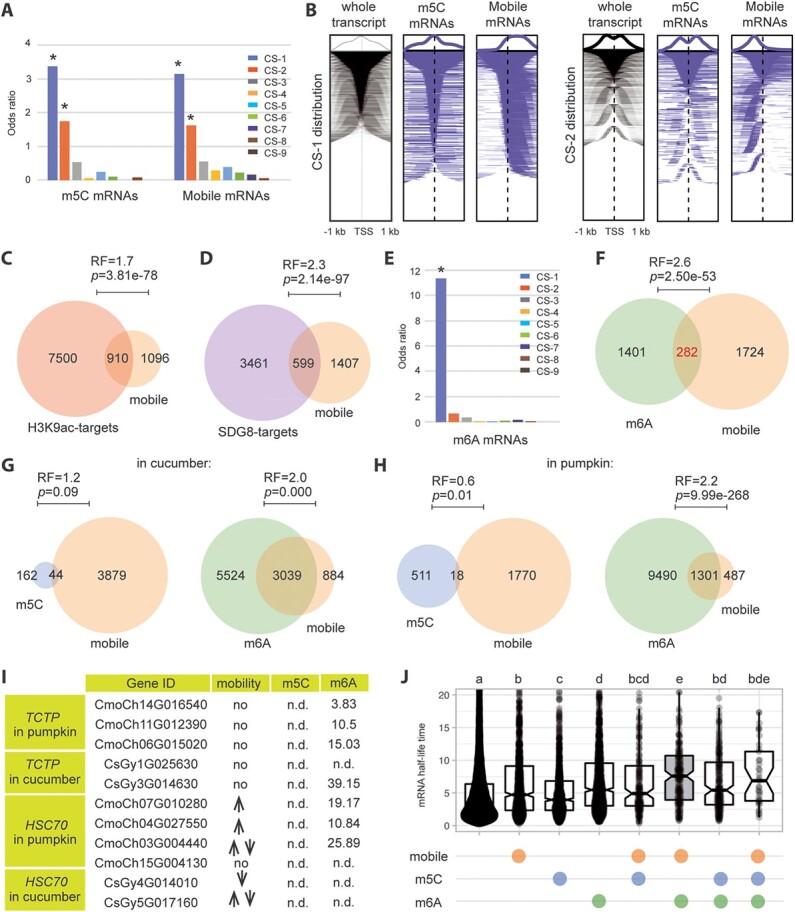
Mobile mRNAs are associated with specific chromatins and marked by m6A. (**A**) Enrichment of m5C and mobile mRNAs in different chromatin states (CS; *, odds ratio > 1 and *p* < 0.05). Mobile mRNA data were collected from various tissues of 5- to 6-week-old Arabidopsis heterografts (col-0/ped-0) exposed to different growth conditions, including full nutrient, nitrogen, and phosphorus starvation. The majority of these data represent shoot-to-root mobility under full nutrient supply [5]. The m5C mRNAs dataset includes information from seedlings grown on MS medium and rosette leaves from soil-grown plants [1]. (**B**) Heat map representing the enrichments of m5C and mobile mRNAs in CS-1 or CS-2. (**C, D**) Overlap between mobile mRNAs and different sets of genes, e.g., H3K9ac-targets (**C**), SDG8-targets *(**D**).* H3K9ac data were generated in seedlings germinated and grown 5 days in dark followed by exposure to 6 hours of light before harvest [6], while SDG8 targets were obtained from 2-week-old seedlings [7]. (**E**) Enrichment of m6A mRNAs in different chromatin states (CS; *, odds ratio > 1 and *p* < 0.05)*.* The m6A mRNAs dataset comes from above-ground tissues of 3 weeks old seedlings [9]. (**F**) Overlap between mobile mRNAs and m6A mRNAs. (**G**) Overlap between mobile mRNAs and m6A/m5C mRNAs in cucumber. (**H**) Overlap between mobile mRNAs and m6A/m5C mRNAs in pumpkin. (**I**) The mobility, m5C/m6A modification of TCTP1 and HSC70.1 orthologs in pumpkin and cucumber. (**J**) mRNA half-life time was determined in mobile, m5C and m6A mRNAs. Different letters indicate significant differences (one-way ANOVA, *p* < 0.05). Chromatin states were defined based on Hidden Markov Model (HMM) and previously defined coordinates were extracted [4]. Transcription Start Sites (TSS) were obtained from www.arabidopsis.org. An array was constructed for each region of interest. The GenomicRanges R-package was used to match the genes in each set with a specific chromatin state. The enrichment of chromatin states was assessed using odds ratios (OR) and False Discovery Rate (FDR)-adjusted p values were used to determine the statistical significance of OR. Statistical comparisons of different ORs were performed through Fisher's exact test, with a significance threshold set at *p* < 0.05. The representation factor (RF) and *p* value for overlapped genes were calculated using the analysis tool from nematode bioinformatics. In the analysis, a background of 31 559, 22 324, 32 205 genes were used for Arabidopsis, cucumber and pumpkin, respectively. An odds ratio or representation factor (RF) exceeding 1 indicates an enrichment or overlap greater than what would be expected based on the proportion of query gene set (e.g., mobile mRNAs) and non-query gene set (e.g., non-mobile mRNAs) in the genome.

Heat maps depicting the distribution of CS distinguished mobile mRNAs from m5C-modified mRNAs (Fig. 1B). While both categories of mRNAs exhibited an association with CS-1, the enrichment of CS-1 in mobile mRNAs primarily occurred within the gene bodies rather than at the transcription start site (TSS). This shift in the distribution of CS-1 prompted us to investigate whether mobile mRNAs are regulated by gene-body specific histone marks within CS-1, such as H3K9ac and H3K36me3. Notably, mobile mRNAs exhibited significant overlap with genes modified by H3K9ac [6] (Fig. 1C) and H3K36me3 via SDG8 [7] (Fig. 1D). Similarly, the CS-2 enrichment of mobile mRNAs displayed a more pronounced enrichments before transcription start sites (TSS). This observation suggested an enrichment of the TSS-associated marker such as H3K4me3 and a lack of gene body histone marks such as H3K27me3. Interestingly, it was reported that m5C peaks were notably enriched in chromatin regions lacking the H3K27me3 repressive histone mark [3]. These findings collectively highlight the unique nature of mobile mRNAs compared to m5C-modified mRNAs. Mobile mRNAs exhibit a strong association with active chromatin regions marked by H3K9ac and H3K36me3. Since H3K36me3 is known to guide m6A modification co-transcriptionally [8], we hypothesized that m6A might also play a significant role in RNA mobility. Importantly, our investigation confirmed that m6A-modified mRNAs [9] were exclusively enriched in CS-1 (Fig. 1E). Moreover, m6A mRNAs were overrepresented within the mobile mRNA group (Fig. 1F).

It is crucial to acknowledge that our datasets originate from diverse plant materials exposed to varying growth conditions, as sourced in the figure legend, which necessitates caution in interpreting our in silico analysis. Considering that the chromatin states are defined based on different tissues, different developmental stages, and different experimental set ups, we had anticipated that any enrichment for tissue-specific or “directionality-specific” mobility of the mRNAs would be diluted out. In other words, the pooled data in chromatin states may lead to false negatives and lower the degree of association between a certain chromatin state and the mobility of RNAs. Yet, we found that CS-1 and CS2 are consistently enriched among the different subsets of mobile RNAs.

To mitigate potential over-interpretation, we specifically chose the CUCUME database, generated within the same experimental framework. The recent CUCUME database (http://cucume.cn) provides comprehensive information on potential mobile mRNAs, along with their m5C and m6A modification status in cucumber and pumpkin, representing a commonly used grafting system. Intriguingly, in pumpkin, there is a limited association between mobile mRNAs and m5C-modified mRNAs. In contrast, m6A modifications are prevalent among mobile mRNAs, particularly in cucumber (Fig. 1G, H).

To gain a deeper understanding, we further examined two well-known Arabidopsis m5C-modified mobile mRNAs, *TCTP1* and *HSC70.1* [1], in pumpkin and cucumber (Fig. 1I). Different orthologs were identified for these genes in the two species, and neither *CsaTCTPs* nor *CmoTCTPs* exhibited mobility. In contrast, *HSC70.1* orthologs in cucumber and pumpkin displayed mobility and were predominantly modified by m6A (Fig. 1I). The mobility of *TCTP1* and *HSC70* underscores the lack of conservation of mobile RNAs across various plant species. This heterogeneity in mRNA mobility between different plant species highlights the complex and species-specific nature of this phenomenon. This leaves us with the question whether there is a conserved aspect underlying mRNA mobility across various plant species.

In the realm of mRNA mobility, a crucial determinant is the chromatin environment. This environment may not be favorable for the transcription of these functionally essential mRNAs, thereby requiring their import from distant tissues. Cell- or organ-specific chromatin landscapes can lead to distinct RNA epigenomes, ultimately determining the direction of their mobility. Additionally, for mRNAs to be efficiently transported, they must remain stable, aligning with the general role of m6A in mRNA stability [9]. Indeed, in contrast to m5C modification, the collaborative action of increased mobility and m6A correlated with longer mRNA half-life time, more strongly than only mobility and mRNA half-life time [2] (Fig. 1J). Another critical requirement for mRNA transportation is that these molecules should not be prematurely engaged by ribosomes for translation within the host cell. Therefore, we wish to emphasize the significance of m6A in facilitating mRNA mobility. m6A has been shown to play a dual role in both activating and inhibiting the translation of its target mRNAs. We suggest that both scenarios are feasible when m6A modifications target mobile mRNAs, implying low translation efficiency within the producing cells and high translation efficiency within the destination cells. Similarly, in neuron cells where mRNAs need to traverse long distances, the interplay between m6A-mediated mRNA stability and mRNA translation efficiency plays a crucial role in orchestrating mRNA localization between the cell body (soma) and neurites [10]. This finding further supports that mRNA mobility is a complex phenomenon that arises from the intricate interplay of mRNA stability and translation efficiency across different species. Looking ahead, the application of cell-type-specific ribosome footprinting techniques holds the potential to provide valuable insights into unraveling the mechanisms underlying mRNA mobility and its regulation. This study may help to better understand the complexities of RNA mobility across different plant species and the varying roles of epigenetics and epitranscriptomics.

Earlier studies proposed an association between m5C and mRNA abundance with RNA mobility in *Arabidopsis thaliana*. In our current letter, we present evidence that the mobile, m5C-modified and m6A-modified mRNAs demonstrate an enrichment in open chromatin regions, signifying active transcription. Furthermore, our in silico analysis demonstrates a robust correlation between mobile mRNAs and m6A modification in Arabidopsis, cucumber and pumpkin, primarily contributing to increased mRNA stability. In future studies, it will be essential to characterize m6A-modified mobile mRNAs, particularly in grafted fruit or vegetable crops. Additionally, investigating the factors that influence the translation of m6A-modified mRNAs and the potential crosstalk between their translation efficiency and mobility is an intriguing avenue for further research.

## Acknowledgements

We thank Dr. Murat Iskar for his feedback, 2020 Marie-Curie fellowship 885864 to Y.D., National Natural Science Foundation of China 32372792 and the 2115 Talent Development Program of China Agricultural University to W.Z., and German Federal Ministry of Education and Research 031B1231B to V.V.U.

## Author contribution

Y.D., V.V.U. and W.Z. initiated the project. Y.D. and V.V.U. performed analysis in Arabidopsis. X.L., Y.C., W.Z. and X.H. performed analysis in cucumber and pumpkin. Y.D. wrote the manuscript. A.B., V.V.U. and W.Z. revised the manuscript.

## Data availability

All data generated for this study are included in the manuscript.

## Conflict of interest statement

The authors declare no conflict of interest.
